# What metabolites are removed by CRRT except creatinine?

**DOI:** 10.1186/s13054-022-04221-8

**Published:** 2022-11-14

**Authors:** Yue Li, Wenhua Liu, Kaijiang Yu, Hongliang Wang, Changsong Wang

**Affiliations:** 1 Department of Intensive Care Medicine, First Affiliated Hospital of Harbin Medical University, Harbin, Heilongjiang China; 2 Department of Intensive Care Medicine, Second Affiliated Hospital of Harbin Medical University, Harbin, Heilongjiang China

*Trial registration* Role and mechanism of intestinal flora in acute kidney in sepsis (ChiCTR2100049312). Registered 29 July 2021.

Continuous renal replacement therapy (CRRT) involves the removal of water and solute by simulating the filtration of normal glomeruli through convection and diffusion [1, 2]. Generally, solutes weighing less than 50,000 daltons can be filtered out [3]; this includes common small molecular substances and medium-sized molecular substances [4, 5]. The loss of inorganic substances in CRRT has received sufficient attention. Some trace elements and minerals, such as K^+^, Na^+^, Ga^2+^, HCO_3_^−^ and glucose, are supplemented during CRRT. However, there is no relevant experimental research on whether organic matter will be lost, in this process, how much will be lost, and how it will affect the body. Thus, the plasma and the ultrafiltrates of AKI patients before and after 24 h of CRRT were investigated in this study based on gas chromatography-tandem time-of-flight mass spectrometry ((GC‒TOF‒MS)) metabolomics to explore the metabolites lost in the plasma during CRRT and the metabolites in the ultrafiltrate.

This study is a prospective study that aims to screen the differential metabolic markers in the plasma and ultrafiltrates of in AKI patients before and after CRRT for 24 h based on GC–TOF–MS nontargeted metabolomics. The standard for staging AKI is based on the Kidney Disease Improving Global Outcomes (KDIGO) definition, which defines injury as an increase in serum creatinine (SCr) by 50% or more and a decline in urine output to < 0.5 mL/kg/hr for 6–12 h. All subjects signed written informed consent before enrollment. This experiment was performed to investigate AKI patients who underwent CRRT in the Department of Critical Medicine of the Second Affiliated Hospital of Harbin Medical University from August 2021 to October 2021. All enrolled patients were treated with a CRRT dialyzer (Prismaflex M100), which was replaced every 24 h. The inclusion criteria were as follows: 1. AKI patients over 18 years old (including 18 years old) and under 65 years old (including 65 years old) that were admitted to the ICU for the first time; 2. patients who underwent CRRT for more than 24 h; and 3. patients who signed informed consent.

A total of 16 AKI patients were enrolled from August 2021 to October 2021. The demographic and clinical data of the participants, including blood samples before CRRT (pre-CRRT), blood samples 24 h after CRRT (post-CRRT), and ultrafiltrate 24 h after CRRT, are detailed in Tables 1 and 2. The differential metabolites in the pre-CRRT group, post-CRRT group, and ultrafiltrate group (Fig. 1) were mainly composed of sugars, fatty acids, amino acids, lipids, carnitine derivatives, and other substances. N-formyl-L-methionine 2, one of the basic units of protein, is the only essential amino acid containing sulfur. It is closely related to the metabolism of various sulfur-containing compounds in organisms, and its absence can cause anorexia, slow growth, no weight gain, kidney swelling, and liver iron accumulation, finally resulting in liver necrosis or fibrosis. Methionine supplementation can be used to prevent and treat chronic or acute hepatitis, liver cirrhosis, and other liver diseases. Lactose 1 is a reducing sugar that is the source of human heat, similar to other sugars. In addition to supplying energy in humans, it also has physiological functions that are different from other sugars. Lactose 1 is not digested and absorbed in the human stomach. However, it can reach the intestine, promote the production of some lactic acid bacteria in the human intestine, inhibit the growth of spoilage bacteria, and contribute to the peristalsis of the intestine. Concurrently, the production of lactose 1 is conducive to the absorption of calcium and other substances. Lysine is one of the essential amino acids of the human body. It can promote human development, enhance immune function, regulate human metabolic balance, and improve the function of the central nervous system. Cystine releases sulfuric acid and increases the detoxification function of the whole metabolic system when metabolized. Additionally, it will assist the supply of insulin, promote cell redox, make liver function vigorous, enhance leukocyte proliferation, and prevent the development of pathogens. These substances beneficial to the body were detected in the CRRT waste liquid bag. If they can be actively supplemented, they may have a positive effect on the body.

**Table 1:** Characteristics of CRRT patients in ICU

Characteristics	Pre-CRRT* n* (16)	Post-CRRT* n* (16)	*P*
*T*, °C	37.03 ± 0.79	36.50 ± 0.30	0.0178
HR, bmp	114.25 ± 26.25	76.63 ± 8.34	< 0.0001
MAP, mmHg	71.79 ± 29.31	97.63 ± 8.34	0.001
RR, bmp	23.50 ± 8.18	14.94 ± 2.29	< 0.0001
PH	7.36 ± 0.17	7.13 ± 0.04	< 0.0001
Lactic acid, mmol/L	4.16 ± 3.66	1.90 ± 0.76	< 0.0001
Serum glucose, mmol/L	6.72 ± 1.65	6.11 ± 1.00	0.107
Leukocyte, × 10^9^/L	14.04 ± 6.01	12.98 ± 3.21	0.2688
Platelet, × 10^9^/L	142.94 ± 99.91	115.31 ± 55.20	0.1704
TBil, mg/dl	57.09 ± 54.57	52.22 ± 44.21	0.0031
Crea, μmol/L	516.19 ± 160.50	325.44 ± 100.22	0.0002
Urea, mmol/L	23.02 ± 12.28	20.32 ± 10.83	0.2572
APACHE-II	25.75 ± 2.41	24.25 ± 1.48	0.0211
SOFA	16.31 ± 2.41	13.25 ± 1.53	0.0002

*T* temperature, *HR* heart rate, *MAP* mean arterial pressure, *RR* respiration, *TBil* total bilirubin, *APACHE-II* acute physiologic assessment and chronic health evaluation II, *SOFA* sequential organ failure assessment, *ICU* intensive care unit

*P* < 0.05 was considered statistically significant

**Table 2:** Characteristics of CRRT patients in ICU

Characteristics	Number and Frequency
Age, y	57 ± 15
Sex, M	10(62.5%)
BMI, kg/m^2^	23.88 ± 3.76
BMR, kJ/(m^2^ h)	− 2.5 ± 11.67
Medications Antibiotic Seralbumin	16 3
Nutritional Support(EN/TPN/NPO)	11/3/2
Insulin therapy	12(75%)

*M* male, *BMI* body mass index, *BMR* basal metabolic rate (Cale), *EN* enteral nutrition, *TPN* totalparenteral nutrition, *NPO* non-peros, *Medications* All drugs used were calculated according to creatinine clearance rate

**Fig. 1:**
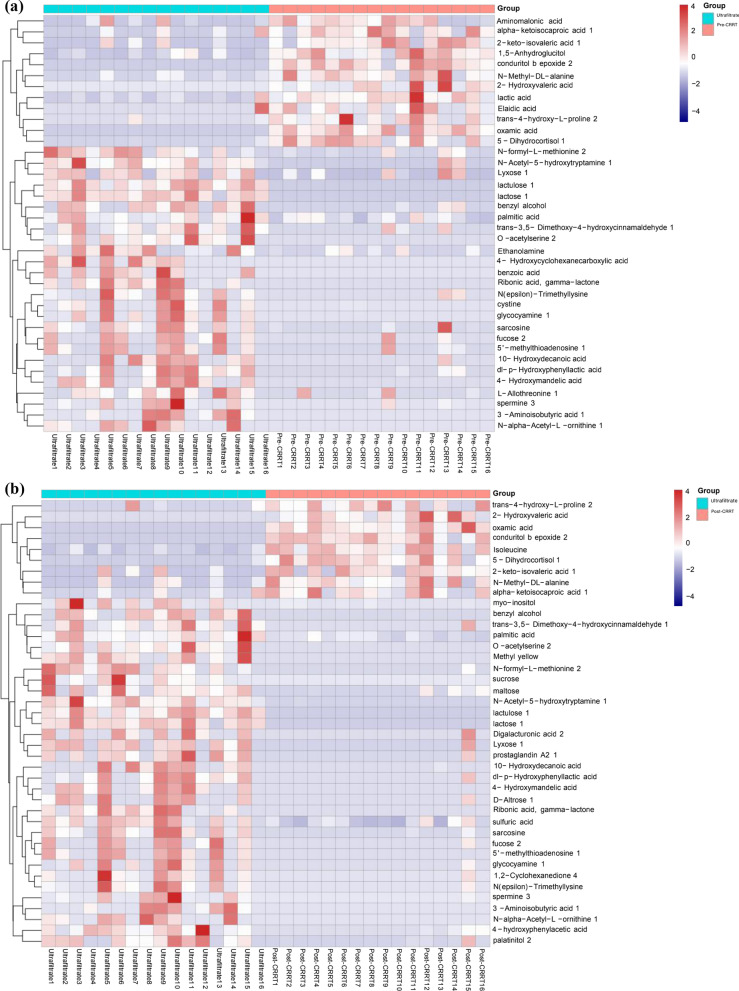

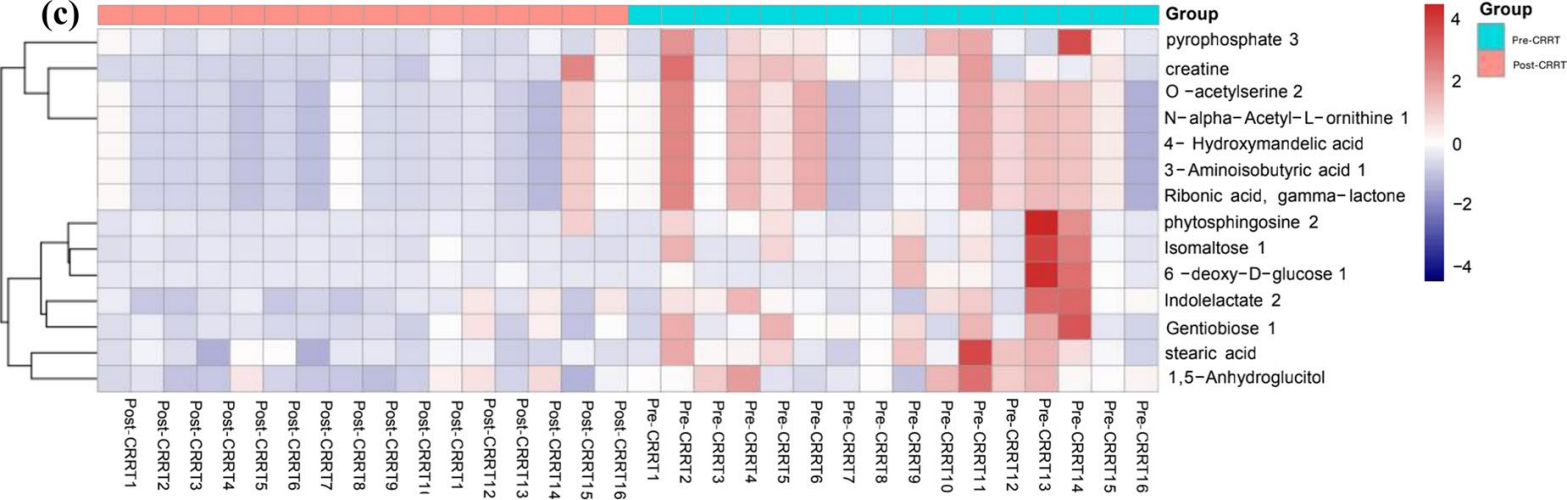
**a** Metabolites differentially expressed in pre-CRRT group and ultrafiltrate group. **b** Metabolites differentially expressed in post-CRRT group and ultrafiltrate group. **c** Metabolites differentially expressed in pre-CRRT group and post-CRRT group

Critically ill patients with AKI, CRRT may contribute to the loss of organic metabolites, such as fatty acids, lipids, carnitine derivatives, and other substances. Loss of these organic metabolites may contribute harm; however, interventional trials evaluated at replacement and supplementation have not been performed. This should be the focus of future clinical trials.

Figure 1 Metabolites differentially expressed in pre-CRRT group, post-CRRT group and ultrafiltrate group. Heat map summarizing level fold changes of significantly altered metabolites in GC‒TOF‒MS data. Red and blue represent higher and reduced concentrations of metabolites in the pre-CRRT group, post-CRRT group, and ultrafiltrate group

## Data Availability

Data sharing is not applicable to this article as no datasets were generated or analysed during the current study.

## References

[1] Bellomo R, Baldwin I, Fealy N (2002). Prolonged intermittent renal replacement therapy in the intensive care unit. Crit Care Resusc.

[2] Huang Z, Letteri JJ, Clark WR, Ronco C, Gao D (2008). Operational characteristics of continuous renal replacement modalities used for critically ill patients with acute kidney injury. Int J Artif Organs.

[3] Sold M (1990). Extrarenale Eliminationsverfahren bei akutem Nierenversagen [Extrarenal elimination procedures in acute kidney failure]. Anaesthesist.

[4] Naorungroj T, Serpa Neto A, Murugan R, Kellum JA, Bellomo R (2021). Continuous renal replacement therapy: the interaction between fluid balance and net ultrafiltration. Am J Respir Crit Care Med.

[5] Murdeshwar HN, Anjum F. Hemodialysis. 2021 Dec 8. In: StatPearls [Internet]. Treasure Island (FL): StatPearls Publishing; 2022. (**PMID: 33085443)**.

